# Automated detection of acute promyelocytic leukemia using an ADVIA 2120i

**DOI:** 10.1111/ijlh.13044

**Published:** 2019-05-03

**Authors:** Daniel Mark Gleghorn, Jan van den Boogaart, Graham Gibbs

**Affiliations:** ^1^ Cambridge University Hospitals NHS Foundation Trust Cambridge UK; ^2^ Siemens Healthcare Nederland B.V. Den Haag Netherlands; ^3^ Siemens Healthcare Ltd Frimley UK

Sir,

Acute promyelocytic leukemia (APL) is a medical and hematological emergency and yet recent studies have shown that early death rates still remain high despite treatment with all‐*trans* retinoic acid (ATRA).^1^, ^2^, ^3^, ^4^

Almost all cases of APL have a diagnostic chromosomal translocation t(15;17),^5^ fusion gene transcript (PML‐RARα),^6^ and distinctive immunophenotype.^7^, ^8^ However, not all hospitals have onsite access to these facilities, the initial diagnosis of APL still remains very much a clinical and morphological decision, and therapy with ATRA may have to be initiated before the final diagnosis is confirmed.^6^ To complicate things further, the microgranular variant form of APL may present with monocytoid‐like morphological features and other subtypes of AML (eg, monoblastic) may also present with coagulopathy.^6^, ^9^, ^10^

Skill levels within routine diagnostic laboratories may be variable, especially in multidisciplinary settings where the ability to “raise the alarm” quickly to clinical staff can be vital. Here, we describe the first prospective evaluation of a novel APL flag for use with ADVIA 2120i hematology analyzers.

The APL flag was developed using data from a test set of 26 confirmed cases of APL and 20 926 non‐APL controls using % immature granulocytes (IG) and %blasts as discriminators (Figure 1).

**Figure 1:**
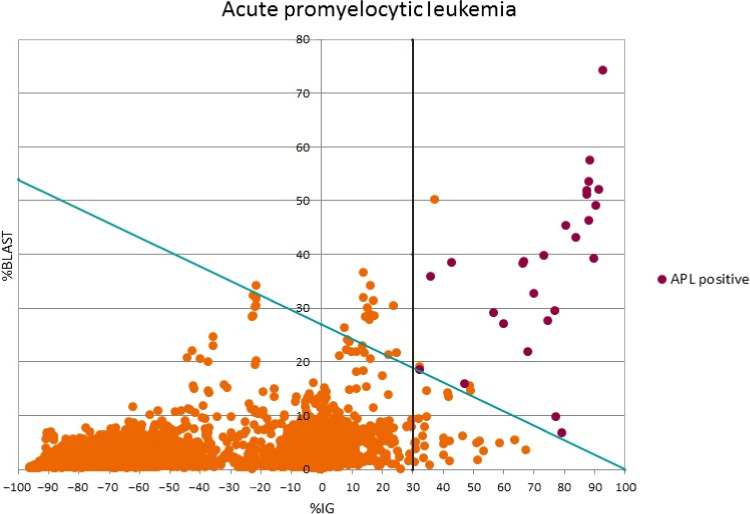
The graph demonstrates the optimum cutoffs for %blasts and %IG for discriminating APL from non‐APL patients. Mathematical functions of these were used to create the APL flag [Colour figure can be viewed at wileyonlinelibrary.com]

Two mathematical functions were then derived in order to create the APL flag: 1) %IG > 30% and 2) %BLAST = −0.27*%IG + 27 to create the rule IF %BLAST + 0.27%IG > 27 AND %IG > 30 THEN APL (Table 1).

**Table 1:** Editing the ADVIA 2120i Data Manager for creation of APL flag (use <Customize>, <System Setup>, <Tools Modify>, and <Test Dictionary>)

Test code	Description	Formula (true = 1)	Host number
%PMN	%PMN	Test number 114 if not already defined	604
%BLAST	%BLAST	Test number 127 if not already defined	605
%IG	%NEUT+%EOS‐%PMN	[%NEUT]+[%EOS]‐[%PMN]	606
APL1	%IG > 30%	0.500*([%IG]+69.90)/100.0	603
APL2	%BLAST+0.27*%IG > 27%	0.500*([%BLAST]+0.27*[%IG]+72.90)/100.0	602
APL	APL positive	0.500*([APL1]+[APL2]+0.900)/2.000	601

For all tests, “auto request” should be selected and the number of decimals set to 1. Host number is not critical; it is only used by the host to address which tests are requested, but the number must be between 0 and 999. If the number used already exists, there will be a warning message and an alternative should be chosen. For the test APL set the Review and Panic Range: “Low” to 0 and “High” to 0.6. In that case, the APL test (or flag) will be highlighted in red.

The APL flag was set up on each of five ADVIA 2120i hematology instruments and captured by the laboratory middleware (CentraLink—Siemens Healthcare Ltd) at Addenbrooke's Hospital, Cambridge University Hospitals NHS Foundation Trust, United Kingdom. All calibration and quality control processes were performed in accordance with the manufacturer's operating instructions.

Data were collected from 418 668 routine blood counts performed between July and December 2018. Duplicate blood counts were removed as some of the patients triggering the APL flag were bled daily.

The diagnosis of APL was confirmed by fluorescence in situ hybridization (FISH) for the PML‐RARα t(15;17) rearrangement, karyotyping, and molecular genetics showing detection of a PML‐RARα fusion gene.

Statistical analysis was performed using Analyse‐it for Microsoft Excel (version 2.30) using cutoffs ranging from ≥0.5 to ≥4.5. With the lowest cutoff observed for positivity of ≥0.7, 63 out of 418 668 blood counts triggered the APL flag (0.015%). Six of these were true positives, and 57 were false‐positive results. There were no false negatives. The optimum cutoff (whereby no true‐positive cases were missed) was shown to be ≥1.0. Using this cutoff, 16 out of 418 668 blood counts triggered the APL flag (0.003%). All six true positives were flagged and ten were false positives. At this cutoff, sensitivity was 100% and specificity was 99.9%, with a positive predictive value (PPV) of 37.5% and negative predictive value (NPV) of 100%.

Early detection is vital for improving patient outcomes. Using the optimum cutoff of ≥1.0, all cases of true APL were flagged. The remaining ten cases were all hematological malignancies that would also require clinical review. In this study, the ten false‐positive cases were clinically and morphologically distinct from APL. Peripheral blood cytopenia is always a concern with APL but the APL flag was shown to work across a wide range of WBC from 0.80 × 10^9^/L to 82.8 × 10^9^/L in this study.

This is the first reported prospective study of the clinical performance of this APL flag in a routine diagnostic laboratory using unselected patients. Further studies are needed to fully evaluate the APL flag with a larger patient cohort including patients with other acute hematological malignancies.

## COMPETING INTEREST

The authors have no competing interests.
